# Supplementary material for: Flux-based hierarchical organization of Escherichia coli’s metabolic network
Source: PLoS Comput Biol. 2020 Apr 20;16(4):e1007832. doi: 10.1371/journal.pcbi.1007832 (PMC7192501; doi:10.1371/journal.pcbi.1007832)
Supplement: Attachment — Submitted filename: response_letter.pdf [file pcbi.1007832.s003.pdf]

Comments to the Authors:

Reviewer #1: The authors investigate the broad field that biological networks across scales exhibit hierarchical organization that may constrain network function. They find that metabolic networks show a hierarchy of reactions based on a natural flux ordering that holds for every steady state, even reflected in experimental measurements of transcript, protein and flux levels of Escherichia coli under various growth conditions as well as in the catalytic rate constants of the corresponding enzymes.

This is nice and well documented here both by the formal analysis as well as the public experimental data used for validation of these findings and a nice analysis.

We would like to thank the reviewers for the positive evaluation of our study.

Comments:
--What should be discussed somewhat more is the direct implications of the results and possible explanations:

(1.1) Certainly, there is some input from resource partitioning and there is fine-tuning of enzyme levels in E. coli and of course it has to respect the constraints imposed by the network structure at steady state.

We do not think that enzyme levels must, *per se*, respect the constraints imposed by the metabolic network at steady state. In fact, this is one of the questions we pose in this study: do the expression levels of enzyme-coding genes, enzyme levels and enzyme activities respect the flux-order relation imposed by the network? In our results, we show that enzyme levels only partially respect the flux order relation. We also show that in certain conditions, enzymes with higher metabolic costs (of synthesis) tend to better follow the flux order relation. We then provide a plausible explanation for this observation, i.e., that to some extent enzyme levels are regulated to agree with the flux order relation to avoid overexpression of unneeded enzyme.

(1.2) However, all this may even more be the result of other more general factors, in particular the evolution of enzyme networks or just the result of fitness optimization, in particular as E.coli is metabolically so active and broad.

We do not understand what the reviewer means by "evolution of enzyme networks" (perhaps evolution of metabolic networks or evolution of protein-protein interaction networks?). Regarding fitness optimization, we do agree with the reviewer’s point. Certainly, regulating gene expression to reduce metabolic costs is evolutionary advantageous in a normal scenario in which nutrients and energy are scarce. We think that both regulating enzyme levels to match the flux order relation as well as tuning of kcat values to match the order relation may be a result of fitness optimization throughout evolution to minimize cellular costs.

(2.1) I agree that reactions in upper layers of the hierarchy impose an upper bound on the flux of the reactions downstream, however, it would be good if the authors can show a nice concrete example on how the hierarchical organization of metabolism due to the flux ordering has direct applications in metabolic engineering, for instance in plants. Again, there is no doubt that you can do metabolic engineering by constraint-based modelling, but it would be good if the authors can clarify how then

the insight in the natural flux ordering presents then a handle to do something novel which is not already obvious from e.g. calculating the flux modes according to standard techniques.

In Section 2.7 we provide one such possible application, i.e., identifying essential reactions which are also flux-ordered with the biomass reaction. As discussed in Section 2.7, the difference is that essential reactions which are also flux-ordered affect flux through the biomass reaction in a continuous manner, while essential reactions which are not flux-ordered affect biomass production in a binary (on/off upon a threshold value) manner. Hence, identifying essential reactions which are flux-ordered with the biomass reaction could allow fine tuning of reaction flux to control biomass production.

The same argument is valid for any other target reaction, which is the main biotechnological application. For instance, lowering the flux through a process of interest can be done by lowering the fluxes (e.g. by knock-down) of reactions in the upper levels of the flux ordering DAG. Furthermore, in contrast to flux modes, which are difficult to determine, the flux ordering DAG is tractable on large-scale networks.

Finally, our study is not an applied, biotechnological study. Rather, we describe a property of metabolic networks, the flux order relation, analyze this property in a specific example, E. coli growing under three carbon sources, and then show that diverse data sets follow to a varying extent the flux order relation. We think that these results merit a study on its own. Our comments in the paper about a possible biotechnological application were meant as a suggestion, and further studies are needed to pinpoint the applications of the flux order relation for a biotechnological application, beyond those noted for modulating growth.

Reviewer #2: In their manuscript "flux-based hierarchical organization ..." the authors propose a new method for computing a hierarchy among metabolic reactions.

Employing the framework of steady-state fluxes obtained via LP from genome-scale metabolic models, the criterion for ranking reactions is flux ordering. The authors compare the resulting hierarchy with categories derived from metabolic pathways, with flux measurements and with regulatory information.

I find the manuscript suitable for PLOS CB. However, I have a few concerns and comments, which the authors should address.

(1.1) *From my perspective the literature around the hierarchical organization of metabolic systems (and biological systems in general) should be cited on a broader level. For example in [Yu, H., & Gerstein, M. (2006). Genomic analysis of the hierarchical structure of regulatory networks. Proceedings of the National Academy of Sciences, 103(40), 14724-14731] the distribution of gene attributes across hierarchical levels has been studied (which is also the strategy employed in the present manuscript). Also, as the authors point out in the introduction, the hierarchical organization of metabolic systems can be assessed on the level of metabolites, as well as on the level of reactions. Examples for the former, which the authors may consider to cite are: [Matthäus, F., Salazar, C., & Ebenhöh, O. (2008). Biosynthetic potentials of metabolites and their hierarchical organization. PLoS Computational Biology, 4(4), e1000049], [Matthäus, F., Salazar, C., & Ebenhöh, O. (2008). Biosynthetic potentials of metabolites and their hierarchical organization. PLoS Computational Biology, 4(4), e1000049].*

We thank the reviewer for pointing us to these relevant references. They are cited now in the introduction.

(1.2) *Regarding the comparison with transcriptional regulation, I regard the findings from [Shlomi, T., Eisenberg, Y., Sharan, R., & Ruppin, E. (2007). A genome-scale computational study of the interplay between transcriptional regulation and metabolism. Molecular systems biology, 3(1)] to be highly relevant. The authors should compare their results with the 'determined' and 'non-determined' categories from this paper.*

We think that the assumptions and objectives of Shlomi et al. markedly differ from ours and hence a direct comparison with our results is not meaningful. Specifically, Shlomi et al. estimate activity patterns of gene expression and reaction flux under different conditions (by coupling a gene regulatory network to the metabolic network) and evaluate the correlation between these two. In this manner, they find reactions whose activity state is either determined or not by the gene activity state. However, we do not evaluate the correlation between activity states (or absolute values for that matter) between gene expression and reaction flux. Our study poses a narrower question: to what extent is the flux order relation respected by diverse data types which are associated, to a different degree, with reaction flux? We would like to emphasize that data values can follow the flux order relation and yet show a poor correlation with fluxes.

(2.1) I was surprised that the authors do not discuss the fact that metabolic fluxes follow a power-law distribution [Almaas, E., Kovacs, B., Vicsek, T., Oltvai, Z. N., & Barabási, A. L. (2004). Global organization of metabolic fluxes in the bacterium Escherichia coli. Nature, 427(6977), 839-843]. Doesn't this already imply a hierarchical organization?

We thank the reviewer for pointing us to this reference. It is now included in the paragraph where we discuss previous evidence of an ordering of fluxes in E. coli.

"[…] In addition, computational and experimental evidence has indicated that reactions in the core of a metabolic network (e.g. 43 the citric acid cycle) generally carry higher flux than reactions in the network 44 periphery, and that genes associated to the reactions carrying higher flux tend to show decreased evolutionary rates [18]. Moreover, the flux distribution of *E. coli* at steady state and under different experimental conditions is highly inhomogeneous and follows a power-law distribution, where a few reactions carry high fluxes and a majority of reactions carry low fluxes [19]. However, power-law distribution of a single flux distribution does not imply ordering of fluxes over multiple flux states."

We note that Almaas et al. do not provide a characterization of the flux order relation in *E. coli*, nor they consider it. They main finding is that fluxes in E.coli distribute in a non-uniform way (power-law or power-law-like), with few reactions carrying high fluxes and a majority carrying low fluxes. As indicated in the extension of the paragraph, power-law flux distribution does not imply flux ordering over multiple flux states.

(2.2) Doesn't this already imply a hierarchical organization?

We do not think that a power-law distribution of reaction fluxes over different experimental conditions necessarily implies a flux order DAG at steady state, although it is consistent with it. For instance, we show in the Jupyter Notebook, Section 2.3 An illustration of a flux-ordered chain, that a random sample of fluxes follows a power-law-like distribution among the reaction in the ordered

chain. However, identifying reactions with high fluxes in a power-law distribution of fluxes does not suffice to ensure that a flux-order DAG exists at steady state let alone to identify such DAG.

(3) How do the authors motivate their choice of iJO1366 as the genome-scale metabolic model? Wouldn't the more recent model iML1515 from [Monk, J. M., Lloyd, C. J., Brunk, E., Mih, N., Sastry, A., King, Z., ... & Feist, A. M. (2017). iML1515, a knowledgebase that computes Escherichia coli traits. Nature biotechnology, 35(10), 904] been a more plausible choice?

While iML1515 is more recent than iJO1366 we do not think that employing iJO1366 instead of iML1515 in our study invalidates our results. On the one hand, iJO1366 is still a consistent model of E. coli metabolism and has been employed and validated in numerous studies. On the other hand, the differences between iML1515 and iJO1366 which are relevant to our study are minimal. We have run a comparative analysis with memote (https://github.com/opencobra/memote), which shows that the two models score very similar in all tests and that the major difference is the number of reactions/metabolites between the two (see attached html containing memote's report).

Specifically, iML1515 adds 187 reactions with respect to iJO1366. Further, if we only consider reactions with associated data (transcript levels under glucose in this example) this figure gets reduced to 167, and only 72 of these 167 reactions are not blocked at steady state. Therefore, we believe that the impact in the presented results of choosing iML1515 would be low and thus are confident that our results remain valid.

(4) In Section 2.2 the authors study the distribution of pathways across the hierarchical levels. Maybe I missed this point, but shouldn't these percentages be compared to a null model of shuffled macrosystem labels, in order to eliminate (level and system) size effects?

In our study (Section 2.4 of the paper and Section 2.4 of the Jupyter Notebook) we compare the representation of each macrosystem across levels. To this end, we compute the frequency (percentage) of each macrosystem among the reactions in each level. While it is true that the first levels contain more reactions than the last levels, we do not think that the approach proposed by the reviewer is necessary. This is because the set of reactions in each level of the DAG is not a random sample of a hypothetical population of reactions in the level, but rather the population itself, i.e., there are no more reactions in each level of the DAG that the ones identified. Therefore, there is no need to correct for the stochasticity involved in random sampling with different sizes.

We have modified the text slightly to further clarify this point.

(5) I have not understood the argument in Section 2.5.1 regarding the potential artifcact due to the empirical focus of flux data on the metabolic core. Wouldn't the appropriate test for this be to evaluate the flux order graph obtained from the E. coli 'metabolic core model' from [Orth, J. D., Fleming, R. M., & Palsson, B. O. (2010). Reconstruction and use of microbial metabolic networks: the core Escherichia coli metabolic model as an educational guide. EcoSal plus]?

We thank the reviewer for raising this point. As it is explained in Section 2.5.1 the potential artefact is with respect to the small metabolic network employed to generate the flux data evaluated in this study. Hence, it makes sense to run the study using that small network and not a core metabolic network of E. coli which was not employed to generate the flux data. In addition, it is often that fluxes inferred from the small E. coli network are used as constraints in studies with the genome-scale metabolic networks (e.g. recent studies by Saur, Alon, and Rabinowitz' laboratories).

The point was to test whether the flux order relation observed in the flux data was inherited from the small network (experimental network as termed in our study). To this end, we looked for flux-ordered reaction pairs in the experimental network and checked whether these pairs overlapped with the ones found in iJO1366.

(6) Given the scope of comparisons in Sections 2.5.2, I would recommend a correction for multiple testing for the p-values reported here.

The p-values reported in this section are empirical p-values, as explained in Methods, Section 4.8, and are result of a permutation analysis in which gene values were randomly shuffled 10000 times. Hence, a single empirical p-value is computed per data type and condition. Since we do not run multiple tests, i.e., these analyses are not per gene, a correction for multiple testing is not appropriate.

(7) I might have misunderstood the analysis strategy of the authors here, but when they say 'a reaction is connected to another by a directional edge if it carries a greater of equal flux in any steady state', do they mean 'any steady state compatible with the minimum flux through the biomass reaction' or do they literally mean any vertex of the flux polytope?

Exactly, it means any steady state compatible with the flux capacity constraints (i.e., $v_{min} \le v \le v_{max}$) as indicated in Section 2.1 and in more detail in Section 4.2. In this case, we decided to constraint the minimum flux through the biomass reaction to recreate a more plausible scenario in which the metabolic network was supporting growth (most experimental measurements were taken in the exponential growth phase of E. coli).

I suppose it is the former. In this case, however, the results will depend strongly on the choice of this minimal flux to be 95 percent of the maximally feasible biomass production rate (as stated in Section 4.7. Should this be the case, I strongly recommend to study the key results under variation of this parameter.

This is an important point. Further constraining the flux cone $C = \{v: Sv = 0, v_{min} \le v \le v_{max}\}$ only adds new flux-ordered reaction pairs to the already found before adding the new constraint. Therefore, increasing the lower flux bound through the biomass reaction has the potential effect of increasing the number of flux-ordered reaction pairs but including all previous ones. That is, going from a minimum flux through the biomass of, say, 95% of the maximum, to one at the 99% of the maximum creates a superset of flux-ordered reaction pairs. By increasing the number of flux-ordered reaction pairs (specifically those with available data) the statistical results reported in our study could change to some degree. The same argument could be applied to decreasing the minimum flux through biomass, although in this case we would be selecting subsets of flux-ordered reaction pairs (with respect to the ones identified at 95% of the maximum capacity).

It is a tricky issue to establish a minimum flux through the biomass reaction that would better reflect the experimental conditions under which measurements were taken. To solve this issue, we have run additional analysis under different values of this parameter: 0%, 50%, 75%, 90%, 92.5% and 100% of the maximum biomass production. We have updated Section 2.5 with the new results, but focused on the thresholds 92.5% and 100% in the text (all results can be found in the online Jupyter Notebook).
