# Supplementary material for: Flux-based hierarchical organization of Escherichia coli’s metabolic network
Source: PLoS Comput Biol. 2020 Apr 20;16(4):e1007832. doi: 10.1371/journal.pcbi.1007832 (PMC7192501; doi:10.1371/journal.pcbi.1007832)
Supplement: Attachment — Submitted filename: re_response_letter.pdf [file pcbi.1007832.s004.pdf]

Comments to the Authors:
Please note here if the review is uploaded as an attachment.

Reviewer #1: The authors revised their manuscript thoroughly, one question remains:

"as discussed in Section 2.7, the difference is that essential reactions which are also flux-ordered affect flux through the biomass reaction in a continuous manner, while essential reactions which are not flux-ordered affect biomass production in a binary (on/off upon a threshold value) manner. Hence, identifying essential reactions which are flux-ordered with the biomass reaction could allow fine tuning of reaction flux to control biomass production."

This is fair enough, but please discuss why not identifying the essential reactions just by looking at those modes which drop out if an enzyme is inhibited does not do a similar good job (if not at least one mode drops out by inhibition of the enzyme this is not an essential enzyme). I would even think that this method is simpler and allows easy to quantify the more important enzymes (as blocking these inhibits a higher number of modes).

--So as mentioned already regarding the previous version of the manuscript, please make still a bit clearer, best by looking at this remaining comment, why and where your approach is preferable to classical elementary mode analysis and what you really gain by using this more complex method of yours.

We sincerely thank the reviewer for her/his time and for the useful comments that have server to improve the manuscript.

We still think that the main difference with respect to flux modes is the capability to identify reactions which impose a continuous constraint on the flux of others, as opposed to the identification of blocked/non-blocked reactions carried out with elementary flux modes. Additionally, the enumeration of elementary flux modes is still computationally challenging for large-scale networks, due to the rapid increase in modes with network size. Finally, we think that elementary flux mode analysis is of course a valid approach, which has proven useful in multiple studies throughout the years. As commented in the previous revision, we emphasize that our study is not a methodological one, nor is our intention to compete with flux modes analysis in any way. Since, we do not include such statements in the paper, we believe that we do not need to do further modifications to the manuscript.

Reviewer #2: The revised version of the manuscript "Flux-based hierarchical organization of Escherichia coli's metabolic network" (PCOMPBIOL-D-19-01496R1) is, from my perspective, a substantial improvement.

We sincerely thank the reviewer for her/his time and for the useful comments that have server to improve the manuscript.

Regarding the work by Almaas et al. (2004), I believe that the manuscript would benefit from a short comment on power-law flux distributions (as most readers will expect this in a publication titled "Flux-based hierarchical organization ...").

We thank the reviewer for this comment, we have added a line to the intro indicating that Almaas et al found that fluxes in E coli follow a power law distribution

I still disagree with the authors' statement that "empirical p-values" do not require a correction for multiple testing. If I consider all groups of flux-ordered reaction pairs assessed here as an individual hypothesis, I would argue that a large set of hypotheses has been tested (and hence a multiple-testing correction would be required). However, this decision is the responsibility of the authors (they explain quite clearly what they are doing).

We did not state that empirical p-values do not require correction for multiple testing as a general fact. What we stated was that, in our case, we performed a single permutation test and thus produced a single empirical p-value for the whole set of flux-ordered pairs for a given experimental condition. Therefore, we still think that multiple testing does not apply here.
